# SPVD-field: a task-oriented multi-task visual dataset for sweet potato virus disease under real field conditions

**DOI:** 10.3389/fpls.2026.1843877

**Published:** 2026-07-10

**Authors:** Ru Han, Lei Shu, Grzegorz Cielniak, Fan Yang, Siyang Zang, Xiaoyuan Jing, Weihan Song, Qian Zhang

**Affiliations:** 1 College of Smart Agriculture (College of Artificial Intelligence), Nanjing Agricultural University, Nanjing, China; 2 Guangdong Provincial Key Laboratory for Green Agricultural Production and Intelligent Equipment, School of Computer Science, Guangdong University of Petrochemical Technology, Maoming, China; 3 School of Engineering and Physical Sciences, University of Lincoln, Lincoln, United Kingdom; 4 Lincoln Institute for Agri-food Technology, University of Lincoln, Lincoln, United Kingdom; 5 School of Electrical Engineering and Automation, Jiangsu Normal University, Xuzhou, China; 6 Key Laboratory of Sweetpotato Biology and Genetic Breeding, Ministry of Agriculture and Rural Affairs, Xuzhou Institute of Agricultural Sciences in Jiangsu Xuhuai District, Xuzhou, China; 7 Jiangsu Academy of Agricultural Sciences, Nanjing, China

**Keywords:** computer vision dataset, lesion segmentation, plant disease detection, precision agriculture, smart farming, sweet potato virus disease

## Abstract

Sweet potato virus disease (SPVD) is one of the most destructive diseases affecting sweet potato production worldwide, causing severe yield losses and posing a significant threat to food security. Vision-based intelligent diagnosis has emerged as a promising solution for large-scale SPVD monitoring due to its low cost and scalability. However, existing publicly available datasets for SPVD are extremely limited and typically focus on a single task, such as disease classification or lesion segmentation, under constrained imaging conditions. This lack of comprehensive, task-oriented datasets significantly restricts the development, evaluation, and fair comparison of advanced computer vision methods for SPVD analysis. In this study, we present SPVD-Field, a task-oriented multi-task visual dataset suite composed of two independently collected sub-datasets optimized for different computer vision tasks. Rather than constructing a single homogeneous dataset, SPVD-Field is deliberately organized into two complementary task-oriented sub-datasets: SPVD-DET, designed for disease detection with bounding-box annotations, and SPVD-SEG, designed for fine-grained lesion segmentation with pixel-level masks. The two sub-datasets were independently collected using different acquisition protocols optimized for their respective tasks, while sharing a unified semantic definition of SPVD symptoms, crop growth stages, and field environments. SPVD-Field captures substantial real-world variability in imaging scale, viewpoint, illumination, background complexity, and symptom manifestation, reflecting the inherent challenges of fieldbased disease diagnosis. We provide detailed documentation of data acquisition, annotation strategies, and quality control procedures, along with baseline benchmark results for both detection and segmentation tasks to demonstrate the usability and difficulty of the dataset. By offering a structured dataset suite rather than a single-task collection, SPVD-Field aims to support diverse research directions, including detection, segmentation, multi-task learning, and disease severity analysis, and to facilitate reproducible and comparable research in SPVD-related plant phenotyping.

## 1 Introduction

Sweet potato (Ipomoea batatas) is a globally important food and industrial crop, particularly in developing regions, where it plays a critical role in food security and rural livelihoods Jiao et al. (2022); Das et al. (2025). Among the various diseases affecting sweet potato production, SPVD is considered one of the most destructive, as it can cause severe growth suppression, leaf deformation, chlorosis, and significant yield losses. Early and accurate identification of SPVD in field environments is therefore essential for effective disease management and precision agriculture practices.

SPVD symptoms exhibit substantial variability across different growth stages, cultivars, and environmental conditions. Early-stage symptoms are often subtle and may only appear as mild chlorosis, slight vein clearing, or faint leaf deformation, whereas severe infections can lead to pronounced mosaic patterns, leaf narrowing, stunted growth, and significant yield reduction. In practical field environments, symptom expression is further influenced by illumination conditions, leaf occlusion, background interference, and mixed infection status, making early visual diagnosis particularly challenging. Because SPVD can spread rapidly and severely affect sweet potato productivity, timely and accurate field monitoring is critically important for disease management, cultivar evaluation, and precision agricultural decision-making.

With the rapid development of computer vision and artificial intelligence, image-based disease diagnosis has become an increasingly attractive approach for large-scale crop health monitoring Zhang et al. (2024). Compared with traditional laboratory-based diagnostic methods, vision-based techniques offer advantages in terms of cost, deployment flexibility, and suitability for in-field applications. In recent years, a growing number of studies have explored the use of deep learning models for SPVD-related tasks, including disease classification, lesion segmentation, and disease severity assessment Zhang et al. (2025); Méndez-Navarro et al. (2024). Despite these advances, progress in this area remains constrained by the lack of high-quality, publicly available datasets that adequately reflect real agricultural conditions.

Most existing plant disease image datasets, including those related to sweet potato, suffer from several common limitations. First, many datasets are collected under controlled or semi-controlled conditions, with relatively clean backgrounds and limited environmental variability, which can lead to overly optimistic performance estimates and poor generalization to real field scenarios Han et al. (2025); Pandey and Mishra (2024). Second, available datasets are typically designed for a single task, such as image-level classification or pixel-level segmentation, making it difficult to study task interactions or to fairly compare methods across different problem formulations. Third, even when multiple datasets exist for the same disease, they are often collected independently with inconsistent annotation standards and usage protocols, further hindering reproducibility and benchmarking.

In practical agricultural applications, however, SPVD diagnosis is inherently a multi-scale and multigranularity problem. Disease detection focuses on localizing symptomatic plants or leaves within complex field backgrounds, while lesion segmentation requires fine-grained delineation of infected regions to support severity estimation and physiological analysis David et al. (2022); Ding et al. (2025). These tasks impose fundamentally different requirements on image acquisition strategies, annotation granularity, and data quality. Attempting to force a single homogeneous dataset to support all tasks often results in compromises that reduce the effectiveness of the data for each individual task.

To address these challenges, we propose SPVD-Field, a task-oriented visual dataset suite for SPVD research under real field conditions. Instead of aggregating all images into a single dataset, SPVD-Field is intentionally structured into two complementary sub-datasets: SPVD-DET, which targets disease detection using bounding-box annotations, and SPVD-SEG, which targets fine-grained lesion segmentation using pixel-level masks. Each sub-dataset is collected using an acquisition protocol optimized for its specific task, while maintaining a unified semantic definition of SPVD symptoms, crop growth stages, and field environments. This design reflects the practical realities of agricultural data collection and enables researchers to select or combine sub-datasets according to their specific research objectives.

The main contributions of this work can be summarized as follows:

1. We introduce SPVD-Field, a multi-source, multi-task dataset suite for SPVD analysis, capturing realistic field variability in scale, viewpoint, illumination, and background complexity.
2. We provide two task-oriented sub-datasets, SPVD-DET and SPVD-SEG, supporting disease detection and lesion segmentation, respectively, with carefully designed annotation strategies and quality control procedures.
3. We release standardized data splits and baseline benchmark results to facilitate reproducible evaluation and fair comparison of SPVD-related computer vision methods.
4. We discuss potential applications and extensions of SPVD-Field, highlighting its value for future research on multi-task learning, disease severity estimation, and field-deployable plant phenotyping systems.

## 2 Related works

### 2.1 Public plant disease image datasets

The rapid advancement of deep learning–based plant disease diagnosis has been closely associated with the availability of public image datasets. Large-scale benchmark datasets, such as PlantVillage, have played a pivotal role in promoting early research on plant disease classification. These datasets typically provide image-level labels under relatively controlled acquisition conditions, with uniform backgrounds and limited environmental variability Cao et al. (2021). While they have significantly accelerated algorithm development, their applicability to real field scenarios is often limited due to the domain gap between controlled and natural environments.

In recent years, several field-collected datasets have been introduced to address this limitation. These datasets aim to capture realistic variations in illumination, occlusion, background complexity, and disease manifestation Xu et al. (2025); Barkessa (2018). Some of them provide object-level annotations for disease detection, while others focus on pixel-level segmentation of lesions. Such efforts have improved the ecological validity of plant phenotyping research and enabled more practical evaluation of computer vision models under field conditions.

However, most existing plant disease datasets remain task-specific. Datasets designed for classification typically lack spatial annotations, whereas detection or segmentation datasets are often restricted to a single annotation granularity Alencastre-Miranda et al. (2021); Zang et al. (2025). Moreover, few datasets are explicitly organized to support coordinated research across multiple related tasks. As a result, cross-task comparisons and integrated modeling approaches remain underexplored.

### 2.2 Vision-based research on sweet potato virus disease

Compared with major crops such as wheat, rice, or tomato, publicly available image datasets for sweet potato diseases are relatively scarce. Research on SPVD has increasingly adopted deep learning methods for automatic diagnosis, including image-level classification, object detection of symptomatic leaves, and lesion segmentation. Thes Xu et al. (2025) studies demonstrate the feasibility of applying convolutional neural networks and transformer-based architectures to SPVD recognition in field environments.

Nevertheless, most SPVD-related studies rely on self-collected datasets that are not publicly accessible, limiting reproducibility and fair benchmarking. In addition, the majority of existing datasets are tailored to a specific modeling objective, such as plant-level detection or lesion-level segmentation, without considering the broader research ecosystem. Differences in acquisition devices, annotation standards, and data splits further complicate cross-study comparison.

Given the practical importance of SPVD management in agricultural production, the absence of a structured and openly available dataset resource has become a significant bottleneck for the field. There is a clear need for a standardized dataset framework that reflects real field conditions while accommodating different levels of spatial granularity.

### 2.3 Multi-task and multi-granularity datasets in agricultural vision

In agricultural computer vision, disease diagnosis inherently involves multiple spatial scales and problem formulations. Detection tasks focus on identifying the location of infected plants or leaves within complex scenes, whereas segmentation tasks require precise delineation of diseased regions for quantitative analysis, such as severity estimation or phenotypic characterization Tibiri et al. (2019); Deng et al. (2023). These tasks differ substantially in terms of image acquisition strategy, annotation workload, and model design.

Despite this intrinsic multi-granularity nature, few plant disease datasets are deliberately designed to support both detection and segmentation research within a unified framework. Attempts to unify all tasks into a single dataset often lead to compromises in image composition or annotation consistency. Conversely, independently developed single-task datasets may lack semantic alignment, making it difficult to conduct joint studies or transfer learning experiments.

To better reflect the complexity of real agricultural scenarios, there is growing recognition that dataset design should consider task specificity while maintaining semantic coherence across tasks. A task-oriented dataset suite, composed of coordinated but independently optimized sub-datasets, may provide a more practical and scalable solution than a monolithic dataset structure.

## 3 Dataset description

### 3.1 Overall structure of SPVD-field

**Figure 1:**
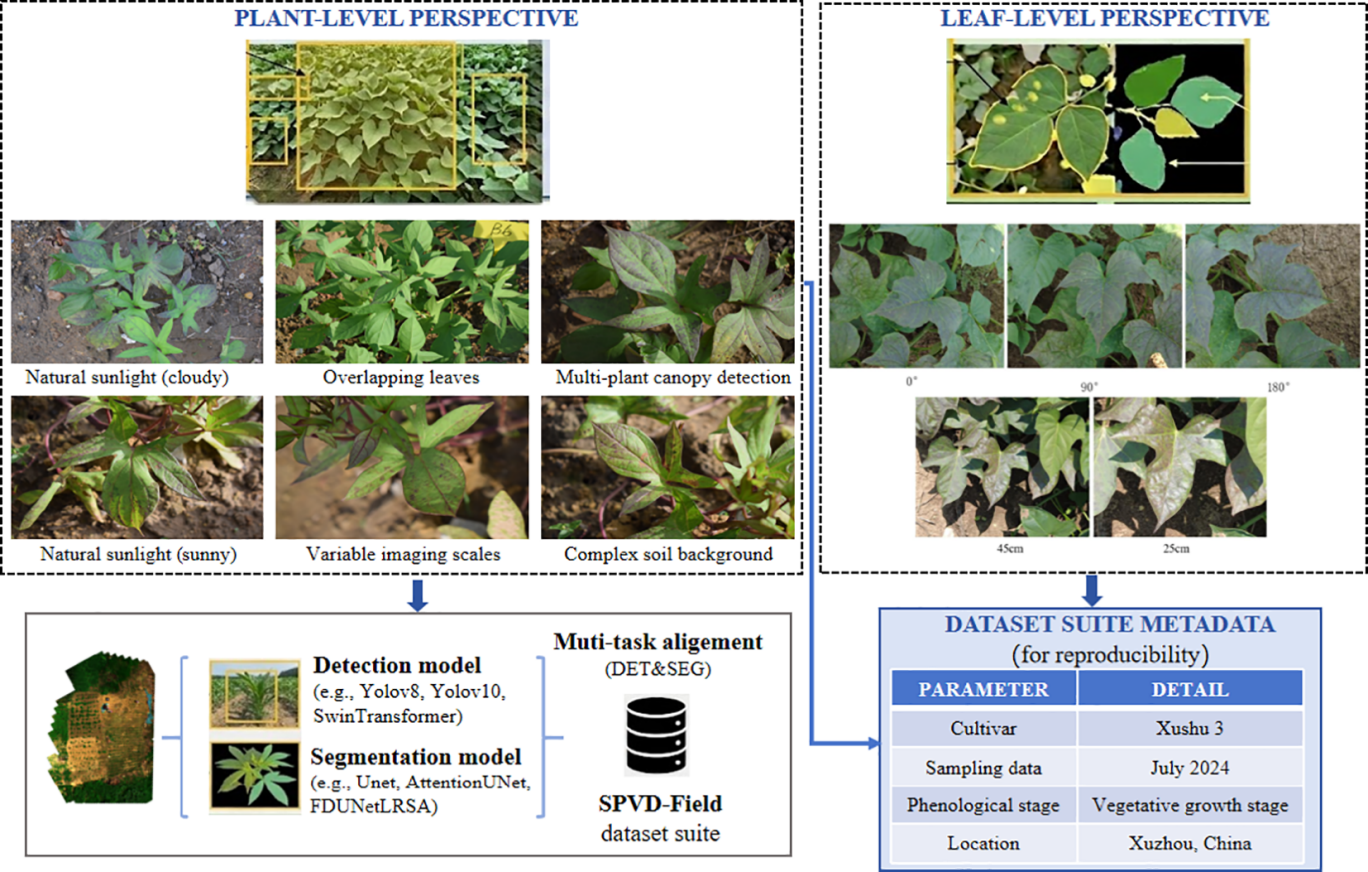
Representative samples of SPVD-field.

SPVD-Field captures substantial real-world variability in imaging scale, viewpoint, illumination, and background complexity. To explicitly illustrate these characteristics, representative sample images under different field conditions are provided in **Figure 1**, including variations in viewing distance, lighting conditions, occlusion, and background clutter. The proposed SPVD-Field dataset suite consists of two task-oriented sub-datasets that were independently collected for different modeling objectives related to SPVD under real field conditions. Rather than merging all images into a single homogeneous collection, the dataset is organized according to task requirements:

- SPVD-DET: designed for disease detection with bounding-box annotations.
- SPVD-SEG: designed for lesion segmentation with pixel-level mask annotations.

**Figure 2:**
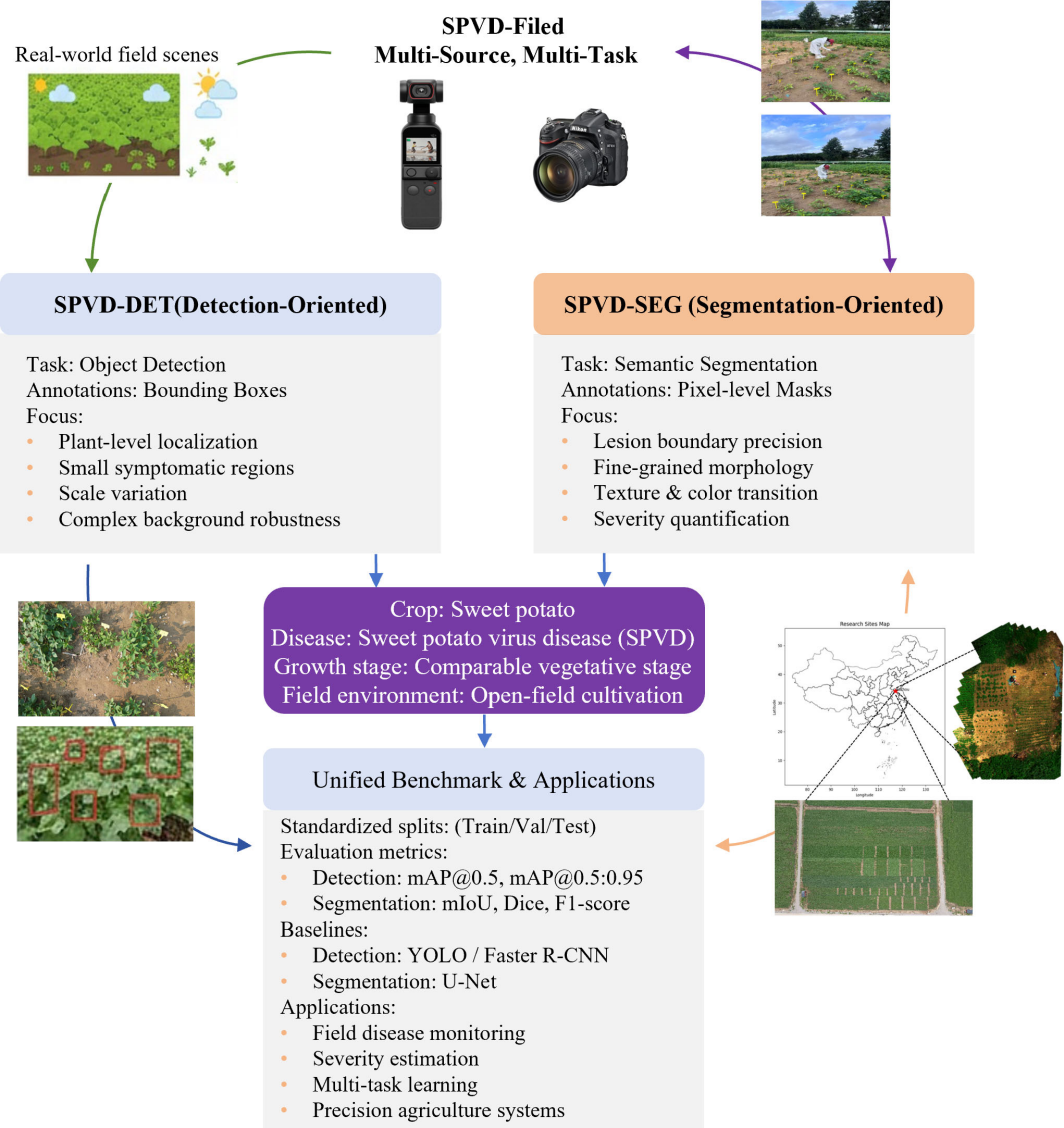
Conceptual overview of the SPVD-field dataset.

As shown in **Figure 2**, It illustrates how the dataset is structured to address disease detection at the plant level and lesion segmentation at the fine-grain level. The figure emphasizes the unified approach for training, validation, and testing across both sub-datasets, using standardized evaluation metrics such as mAP, IoU, and Dice score.

Although the two sub-datasets were constructed in separate studies, they share consistent semantic definitions of SPVD symptoms and were collected from open-field sweet potato cultivation environments. Both sub-datasets aim to reflect realistic agricultural conditions, including natural illumination variations, background complexity, and heterogeneous symptom presentation. A summary of the dataset components is provided in **Table 1**, It shows the variations in training, validation, and testing splits across both detection and segmentation tasks.

**Table 1:** Overall statistics of the SPVD-field dataset.

Attribute	SPVD-DET	SPVD-SEG (Orig.)	SPVD-SEG (Half)	SPVD-SEG (Full)
Task	Object Detection	Segmentation	Segmentation	Segmentation
Original Images	1,264	1,095	1,095	1,095
Augmented Images	1,093	–	1,590	2,491
Total Images	2,357	1,095	2,685	3,586
Train	1,886	876	2,156	2,868
Val	353	164	404	537
Test	118	54	134	180
Annotation Type	Bounding Boxes	Pixel Masks	Pixel Masks	Pixel Masks
Resolution	6000×400/3840×2160	256×256	256×256	256×256

### 3.2 SPVD-DET: detection-oriented sub-dataset

#### 3.2.1 Image acquisition

The SPVD-DET sub-dataset was collected in open-field sweet potato planting areas during the active growth stage. RGB images were captured using handheld imaging devices under natural lighting conditions. No artificial background control or illumination adjustment was applied during acquisition in order to preserve real field variability.

Images were acquired from multiple viewpoints and distances, including both plant-level and leaflevel perspectives. This acquisition strategy intentionally introduced variations in scale, occlusion, and background clutter to simulate realistic monitoring scenarios.

#### 3.2.2 Annotation protocol

SPVD-DET is annotated with bounding-box labels for object detection tasks. The annotation process focuses on identifying symptomatic regions corresponding to SPVD-affected plants or leaves within each image. The bounding-box annotations were created using the LabelImg annotation tool, which is widely adopted for object detection tasks. The annotations were exported in standard YOLO format to ensure compatibility with mainstream detection frameworks.

Annotations were manually generated based on visual symptom characteristics, including leaf curling, chlorosis, mosaic patterns, and growth abnormalities. Each bounding box encloses a visually distinguishable diseased region. The annotation format follows standard object detection conventions (e.g., YOLO or COCO format).

#### 3.2.3 Dataset composition

**Figure 3:**
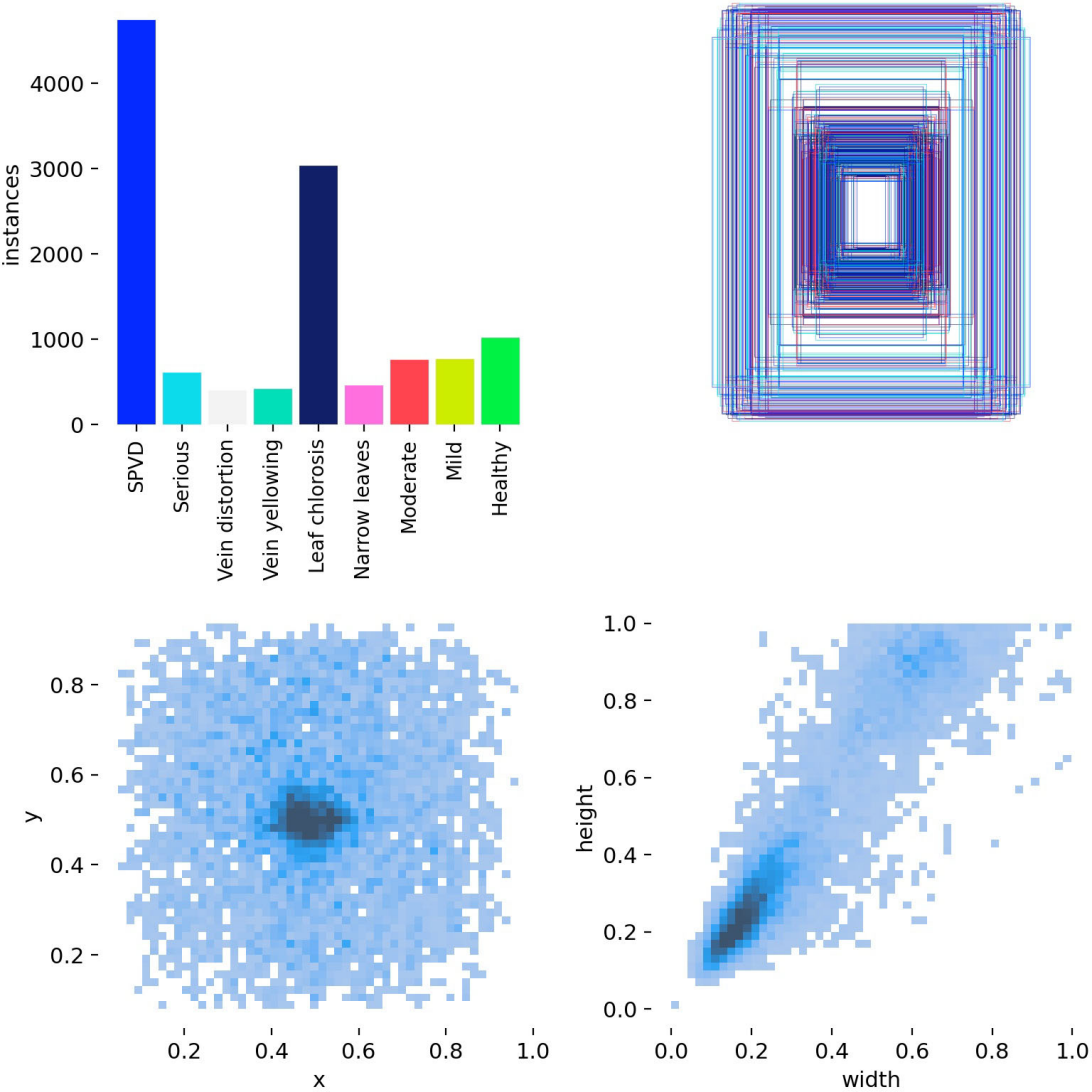
Multi-dimensional analysis of the SPVD-DET dataset: class distribution, bounding box size, and spatial location characteristics.

The SPVD-DET sub-dataset contains a total of 1264 RGB images, with corresponding bounding-box annotations. To better understand the characteristics of the SPVD-DET dataset, we further analyze the distribution of annotated instances, bounding box sizes, and spatial positions. As shown in **Figure 3**, the dataset exhibits a multi-class distribution of disease symptoms, including vein distortion, vein yellowing, leaf chlorosis, narrow leaves, and severity levels such as mild and moderate infection. Among these categories, SPVD and leaf chlorosis contain the largest number of instances, reflecting their prevalence in real field environments.

The bounding box visualization indicates that the dataset contains objects with significant scale variation, ranging from small lesion regions to larger plant-level symptoms. The width–height distribution further confirms the presence of numerous small objects, which increases the difficulty of detection tasks.

Additionally, the spatial heatmap reveals that most disease regions are concentrated around the central region of the image, which corresponds to the typical camera framing used during field data acquisition. However, a substantial number of annotations also appear in peripheral areas, providing diversity in spatial distribution.

This multi-dimensional distribution demonstrates that the SPVD-DET dataset captures realistic disease patterns and provides sufficient complexity for evaluating detection algorithms.The dataset is divided into training, validation, and test subsets according to a fixed split strategy. The images exhibit substantial variability in:

- Object scale (small symptomatic regions to larger affected leaves).
- Background complexity (soil, weeds, overlapping leaves).
- Illumination (cloudy, sunny, shadowed regions).
- Symptom severity levels.

These characteristics make SPVD-DET suitable for evaluating the robustness and generalization capability of detection models under real agricultural conditions.

### 3.3 SPVD-SEG: segmentation-oriented sub-dataset

#### 3.3.1 Image acquisition

The SPVD-SEG sub-dataset was independently collected in field environments during sweet potato growth stages characterized by visible SPVD symptoms. RGB images were captured using handheld or stabilized imaging devices at relatively closer distances compared to SPVD-DET, in order to facilitate precise lesion-level annotation.

All images were acquired under natural field conditions without artificial background isolation. Variations in illumination, leaf overlap, and background interference were intentionally preserved to reflect practical deployment scenarios.

#### 3.3.2 Annotation protocol

SPVD-SEG is annotated with pixel-level masks corresponding to SPVD lesion regions. The annotation process was conducted manually using image annotation tools to delineate the boundaries of symptomatic areas. The pixel-level annotations were generated using the Labelme annotation tool, which supports precise polygon-based mask annotation. Annotators manually delineated lesion boundaries using free-form polygons to accurately capture irregular shapes and fine-grained structures.

Special attention was given to:

- Irregular lesion shapes
- Gradual color transitions between healthy and diseased tissue
- Partially occluded regions

Mask annotations were refined through iterative checking to improve boundary accuracy. In cases where lesion boundaries were extremely ambiguous due to severe blur or lighting artifacts, the samples were excluded to maintain annotation reliability.

The final annotations are provided in standard segmentation formats (e.g., binary mask images aligned with the original RGB images). To ensure the quality and reliability of the segmentation annotations, a multi-stage verification process was conducted. First, all annotated masks were reviewed by experienced annotators to correct obvious boundary inaccuracies. Second, a cross-checking strategy was adopted, where a subset of images was independently re-annotated and compared to evaluate consistency. Finally, ambiguous samples with unclear lesion boundaries were excluded from the dataset to maintain annotation precision.

#### 3.3.3 Dataset composition

**Figure 4:**
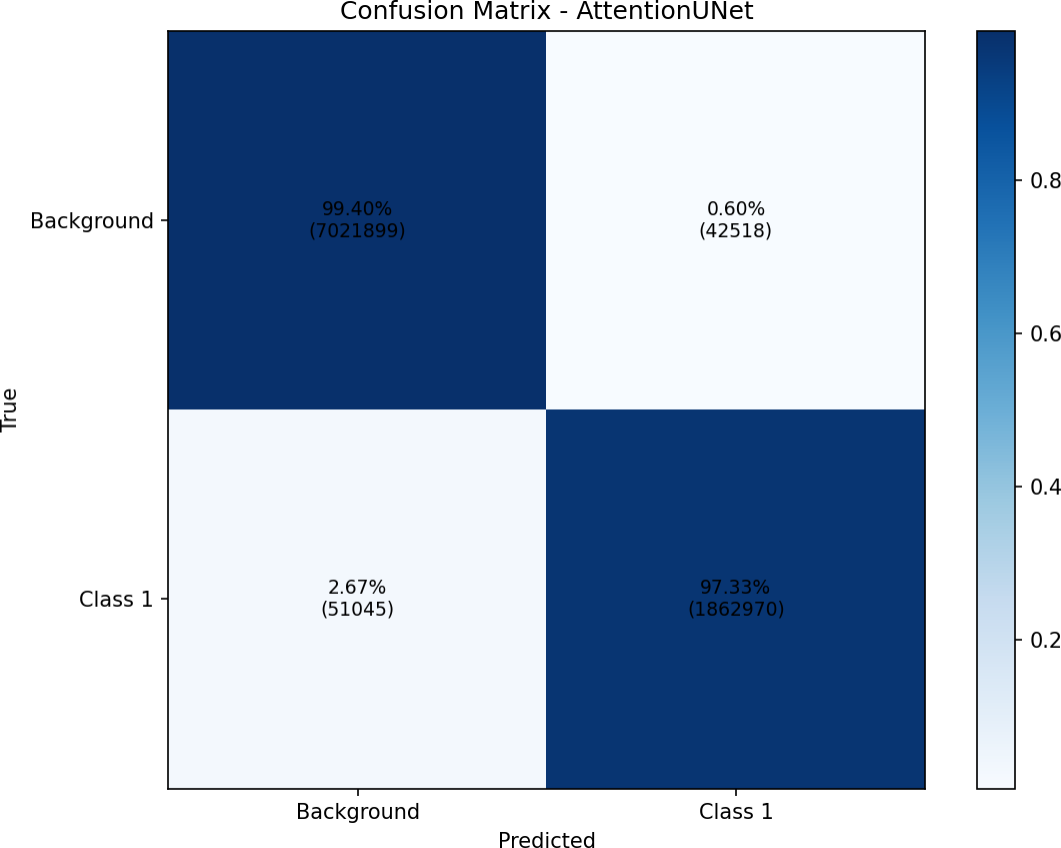
Confusion matrix of the SPVD-SEG dataset.

The SPVD-SEG sub-dataset contains 1095 RGB images, each paired with a corresponding pixel-level lesion mask. The confusion matrix shown in **Figure 4** further illustrates the segmentation performance. The model correctly identifies 97.33% of SPVD lesion pixels, while background classification accuracy reaches 99.40%. Only a small proportion of lesion pixels are misclassified as background, which mainly occurs at ambiguous lesion boundaries. Compared with SPVD-DET, SPVD-SEG emphasizes:

1. Fine-grained lesion morphology.
2. Boundary delineation.
3. Local texture and color variations

This sub-dataset is suitable for evaluating semantic segmentation networks, fine-grained disease analysis, and potential downstream tasks such as lesion area quantification or severity estimation.

### 3.4 Relationship between the two sub-datasets

Although SPVD-DET and SPVD-SEG were collected independently and are not derived from the same image pool, they share:

1. The same crop species (sweet potato).
2. The same disease definition (SPVD)
3. Similar field cultivation environments
4. Comparable growth stages

The separation into two sub-datasets reflects differences in task objectives and annotation granularity rather than semantic inconsistency. SPVD-DET focuses on spatial localization of diseased regions within complex scenes, while SPVD-SEG concentrates on fine-scale lesion boundary delineation.

By organizing the dataset as a coordinated suite rather than a single unified collection, SPVD-Field preserves task-specific data quality while maintaining semantic coherence across sub-datasets.

## 4 Methods

### 4.1 Study area and field data acquisition

The SPVD-Field dataset was constructed from open-field sweet potato cultivation areas located in Xuzhou, China. The dominant cultivar was “Xushu 3,” which is widely cultivated in regional agricultural production. Data collection was conducted during the vegetative growth stage in July 2024, when SPVD symptoms were visually distinguishable.

All images were captured under natural field conditions without artificial lighting or background isolation. Environmental variability, including sunlight intensity, partial shading, soil background, weed interference, and leaf occlusion was intentionally preserved to reflect realistic agricultural deployment scenarios.

### 4.2 Evaluation framework

To systematically assess the usability and research value of the proposed SPVD-Field dataset, we establish a unified evaluation framework covering both object detection and lesion segmentation tasks. The framework aims to provide standardized benchmark protocols that enable reproducible evaluation and fair comparison among different computer vision models.

For the SPVD-DET sub-dataset, the evaluation focuses on the ability of models to localize symptomatic plants or leaves in complex field environments. The performance of detection algorithms is assessed using widely adopted object detection metrics, including Precision (P), Recall (R), and mean Average Precision (mAP) at different Intersection over Union (IoU) thresholds. In particular, mAP@0.5 and mAP@0.5:0.95 are reported as the primary indicators of detection accuracy and localization quality. These metrics are consistent with commonly used evaluation standards in modern detection frameworks such as the YOLO series and transformer-based detectors.

To verify the effectiveness of the dataset for detection research, several representative baseline models are employed, including state-of-the-art YOLO-based architectures and hybrid CNN–Transformer models. These models have demonstrated strong performance in agricultural vision tasks and provide meaningful benchmarks for SPVD detection. Through comparative experiments, the evaluation framework highlights the challenges posed by real-field variability, such as complex backgrounds, illumination changes, and small symptomatic regions.

For the SPVD-SEG sub-dataset, the evaluation focuses on fine-grained lesion segmentation performance. Pixel-level prediction accuracy is measured using Intersection over Union (IoU) and the Dice coefficient, which are standard metrics for semantic segmentation tasks. IoU evaluates the overlap between predicted lesion regions and ground-truth masks, while Dice emphasizes boundary consistency and overall segmentation accuracy. These complementary metrics enable comprehensive assessment of segmentation models in capturing irregular lesion shapes and subtle disease boundaries.

By combining detection and segmentation evaluations within a unified framework, the SPVD-Field dataset provides a multi-granularity benchmark that supports diverse research directions, including object detection, semantic segmentation, multi-task learning, and disease severity analysis. This framework also ensures that experimental results are directly comparable with existing computer vision research on plant disease recognition.

### 4.3 Experimental settings

To ensure reproducibility and fair comparison across different algorithms, all benchmark experiments were conducted under standardized experimental settings for both detection and segmentation tasks.

#### 4.3.1 Dataset splits

The SPVD-Field dataset provides predefined training, validation, and test splits for each sub-dataset. For SPVD-DET, the dataset contains 2,357 images after augmentation, which are divided into 1,886 training images, 353 validation images, and 118 test images. For SPVD-SEG, the dataset includes 1,095 original images with pixel-level annotations, which are split into 876 training images, 164 validation images, and 54 test images. These standardized splits ensure consistent evaluation across different studies and prevent data leakage between training and testing stages.

#### 4.3.2 Detection task configuration

For the detection task, several representative deep learning detectors are adopted as benchmark models, including YOLOv8, YOLOv9, YOLOv10, and the hybrid Swin-SPVDNet architecture. These models were selected because they represent different design paradigms, including lightweight convolutional detectors and CNN–Transformer hybrid networks.

All detection models were trained using RGB images under the same training protocol. Input images were resized to a fixed resolution compatible with the detection framework. Data augmentation techniques such as random flipping, rotation, contrast adjustment, and random cropping were applied to improve model generalization and simulate variations commonly observed in field environments. Training was conducted using the PyTorch deep learning framework, with an adaptive optimizer and learning rate scheduling strategy. The models were trained for a fixed number of epochs until convergence, and the best-performing checkpoint on the validation set was selected for final testing.

#### 4.3.3 Segmentation task configuration

For the segmentation task, several widely used semantic segmentation networks were evaluated, including UNet, Attention-UNet, UNetSmall, SwinUNet, and FDUNet-LRSA. These architectures cover both classical convolutional segmentation networks and transformer-based models, providing a comprehensive benchmark for lesion segmentation research.

All segmentation models were trained using cropped images with a fixed spatial resolution to balance computational efficiency and segmentation accuracy. Standard data preprocessing steps were applied, including normalization and resizing. During training, data augmentation strategies such as random flipping, rotation, and color perturbation were used to enhance dataset diversity and reduce overfitting.

#### 4.3.4 Implementation details

All experiments were implemented using the PyTorch framework and executed on a workstation equipped with an NVIDIA GPU. Model training followed standard deep learning practices, including mini-batch optimization, learning rate scheduling, and early stopping based on validation performance. Evaluation metrics were computed on the held-out test sets to provide unbiased estimates of model performance. Detailed hardware configurations, software environments, and training settings are specified in **Table 2**.

**Table 2:** Hardware configuration and software environment.

Category	Item/specification	Details
Hardware Platform	CPU	Intel(R) Xeon(R) Platinum 8488C @ 3.80 GHz
	GPU	NVIDIA GeForce RTX 4090 (24 GB VRAM)
	RAM	512 GB DDR4
	Storage	NVMe SSD 2 TB
Operating System	OS Version	Ubuntu 22.04 LTS (64-bit)
Software Environment	Programming Language	Python 3.8.16
	Deep Learning Framework	PyTorch 2.1.0
	CUDA Toolkit	CUDA 11.8
	cuDNN Version	cuDNN 8.9.6
	IDE	Visual Studio Code 1.96.0
	Supporting Libraries	NumPy, OpenCV, SciPy, Matplotlib, Albumentations, TorchVision
Training Settings	Training Batch Size	8
	Training Epochs	300
	Mixed Precision	FP32

By adopting consistent experimental configurations and representative baseline models, the SPVD-Field dataset establishes a reliable benchmark for evaluating computer vision algorithms in sweet potato disease detection and segmentation under realistic field conditions.

### 4.4 Evaluation metrics

The evaluation was conducted on two primary tasks: disease detection and lesion segmentation. The evaluation framework includes standard computer vision metrics, including precision, recall, and mean average precision (mAP) for detection, and Intersection over Union (IoU) and Dice coefficient for segmentation. These metrics provide comprehensive assessments of the algorithms’ robustness under varying real-field conditions. The following relevant index equations can be found in Equations 1–6:

• Precision (P): the ratio of correctly predicted positive instances to the total predicted positive instances, defined as

(1) $$\text{Precision}=\frac{TP}{TP+FP}$$

where TP denotes true positives and FP denotes false positives.

• Recall (R): the ratio of correctly predicted positive instances to all actual positive instances, defined as

(2) $$\text{Recall}=\frac{TP}{TP+FN}$$

where FN denotes false negatives.

• F1 Score: the harmonic mean of precision and recall, providing a balanced measure of both, defined as

(3) $$F1=2\times \frac{\text{Precision}\times \text{Recall}}{\text{Precision}+\text{Recall}}$$

• Mean Average Precision (mAP): the mean of average precision scores across all categories, computed as the area under the precision–recall curve for each class, then averaged across all classes. Formally, for N classes:

(4) $$mAP=\frac{1}{N}\sum_{i=1}^{N}AP_{i}$$

where *AP_i_* denotes the average precision of the *i^th^* class. These metrics collectively provide a rigorous and comprehensive evaluation of detection performance, enabling detailed analysis of strengths and limitations across different SPVD symptom categories and environmental conditions.

• Intersection over Union (IoU): The ratio of the intersection of the predicted segmentation mask and the ground truth mask to the union of both, providing a measure of the overlap between them, defined as:

(5) $$\text{IoU}=\frac{\left|A\cap B\right|}{\left|A\cup B\right|}$$

where *A* represents the predicted mask and *B* represents the ground truth mask. The IoU is a value between 0 and 1, with 1 indicating perfect overlap.

• Dice Coefficient: The harmonic mean of precision and recall for segmentation tasks, providing a balanced measure of both, defined as:

(6) $$\text{Dice}=\frac{2\times \left|A\cap B\right|}{\left|A\right|+\left|B\right|}$$

where |*A*| and |*B*| are the areas of the predicted mask and ground truth mask, respectively. The Dice Coefficient ranges from 0 to 1, with 1 indicating perfect overlap between the predicted and ground truth masks.

In this study, we adopt mAP@0.5 as the primary evaluation metric, as it is widely used for baseline benchmarking in object detection tasks and aligns with our goal of providing a foundational dataset for SPVD detection and segmentation research without introducing excessive complexity.

## 5 Dataset verification

To validate the usability and research value of the proposed SPVD dataset, we conducted a series of benchmark experiments covering both object detection and semantic segmentation tasks. Multiple representative deep learning models were evaluated under a unified experimental protocol. The results demonstrate that the dataset supports reliable model training and enables comprehensive evaluation under realistic agricultural field conditions.

### 5.1 Overall performance comparison

To evaluate the effectiveness of the SPVD-DET dataset for object detection tasks, several representative detection models were trained and tested, including YOLOv8, YOLOv9, YOLOv10, and the proposed Swin-SPVDNet. The quantitative results are summarized in **Table 3**. Swin-SPVDNet achieves the best performance across all metrics, reaching 85.1% Precision, 75.6% Recall, 86.8% mAP@0.5, and 76.0% mAP@0.5:0.95, outperforming the other baseline detectors. Compared with the widely used YOLOv8 model, Swin-SPVDNet improves mAP@0.5 by approximately 3.0%, indicating that transformer-based feature modeling can effectively enhance the detection of complex SPVD symptoms under field conditions.

**Table 3:** Performance of different detection models for SPVD-DET.

Model	Precision (%)	Recall (%)	mAP50 (%)	mAP95 (%)
Yolov8	82.1	72.1	83.8	71.5
Yolov9	76.3	**83.1**	82.8	72.3
Yolov10	83.8	72.5	84.9	74.8
Swin-SPVDNet	**85.1**	75.6	**86.8**	**76.0**

Bold values represent the results that are the best among all.

The comparative results demonstrate that the SPVD-DET dataset is capable of supporting the training and benchmarking of modern detection architectures while maintaining sufficient challenge due to small lesion regions, occlusion, and complex backgrounds.

**Figure 5:**
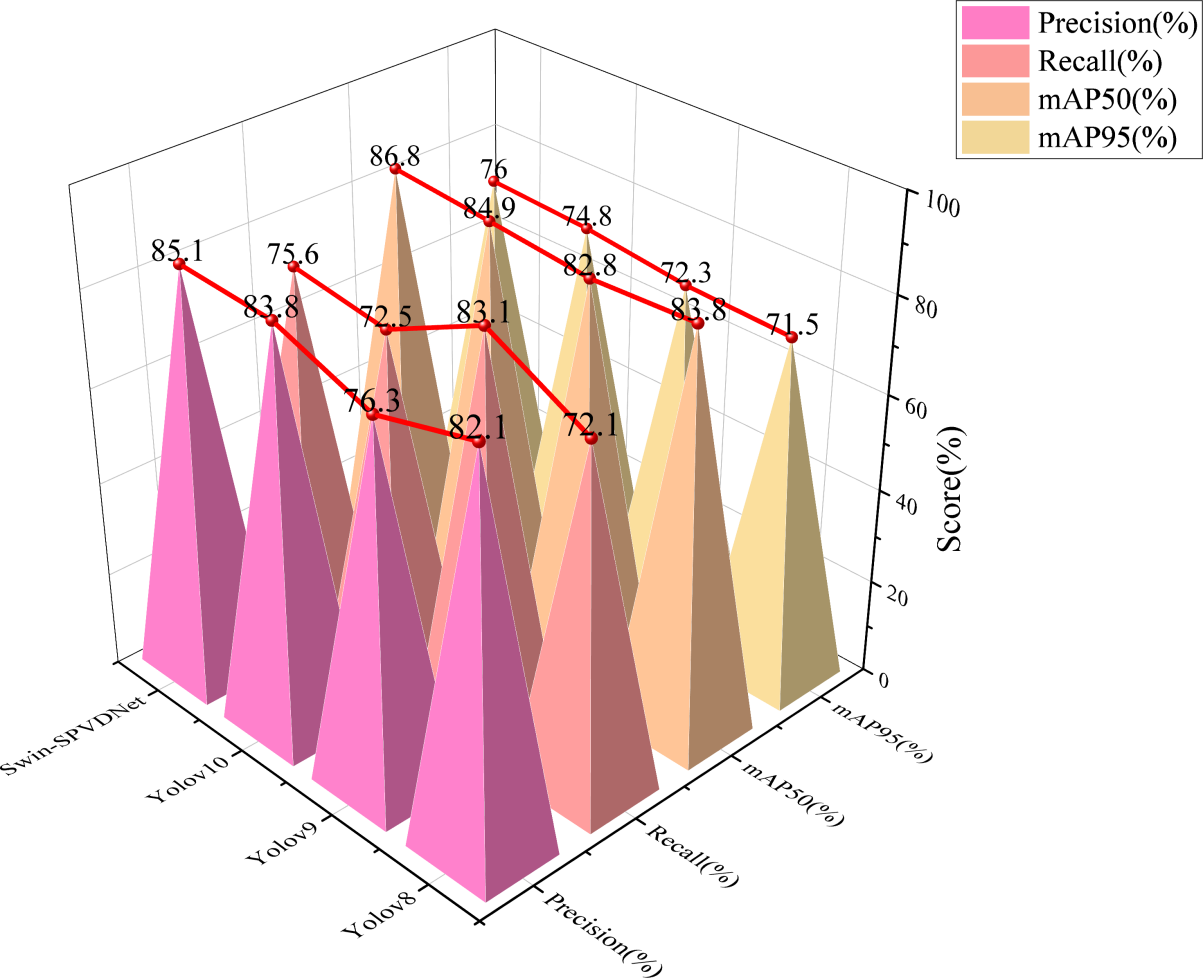
Comparison with other detection models.

As illustrated in **Figure 5**, Swin-SPVDNet consistently achieves higher scores across all evaluation metrics, particularly in Precision and mAP, indicating its superior capability in capturing fine-grained disease characteristics. Meanwhile, YOLOv9 achieves the highest recall, suggesting a stronger ability to identify potential disease regions, albeit with slightly reduced localization accuracy.

**Figure 6:**
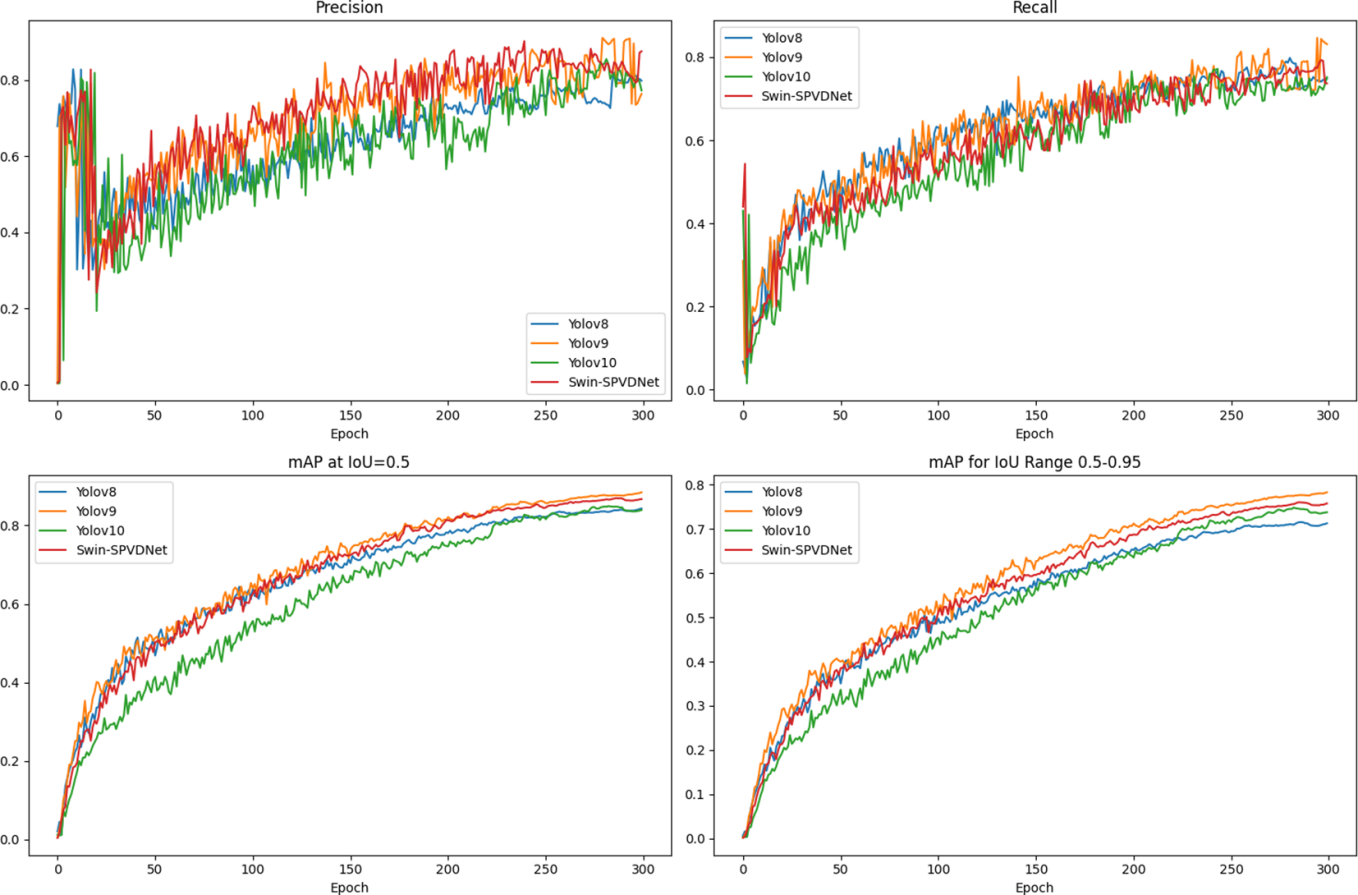
Training performance comparison of SOTA object detection models.

To further analyze the learning behavior of different detection models on the SPVD-DET dataset, the training curves of Precision, Recall, mAP@0.5, and mAP@0.5:0.95 across epochs are presented in **Figure 6**. we can see that all models exhibit a similar convergence trend. The evaluation metrics increase rapidly during the early training stage and gradually stabilize after approximately 200 epochs, indicating stable optimization on the SPVD-DET dataset.

Among the compared models, Swin-SPVDNet and YOLOv9 achieve the best overall performance, reaching mAP@0.5 values close to 0.88 after convergence. Swin-SPVDNet maintains higher precision in later epochs, demonstrating stronger capability in distinguishing SPVD symptoms from complex field backgrounds. YOLOv9 shows slightly higher recall, indicating stronger sensitivity in detecting potential disease regions.

For the stricter mAP@0.5:0.95 metric, Swin-SPVDNet remains competitive throughout training, reflecting its improved localization accuracy across different IoU thresholds.

Overall, the results demonstrate that the SPVD-DET dataset supports stable training for modern detection architectures and provides sufficient complexity for benchmarking different models.

In addition to quantitative metrics, we further analyze the dataset performance through training dynamics and segmentation results. Several representative semantic segmentation models were evaluated on the SPVD-SEG dataset, As shown in the **Table 4**.

**Table 4:** Comparison with other segmentation networks.

Model	Params	FLOPs	IoU	Dice	F1	Accuracy
UNet	31.0M	95.42	0.94	0.97	0.97	0.98
AttentionUNet	34.8M	103.1	0.94	0.96	0.96	0.98
UNetSmall	4.3M	93.31	0.93	0.96	0.96	0.98
SwinUNet	27.1M	98.2	0.88	0.93	0.93	0.97
**FDUNetLRSA**	∼35M	**72.1**	**0.94**	**0.97**	**0.97**	**0.99**

Bold values represent the results that are the best among all.

**Figure 7:**
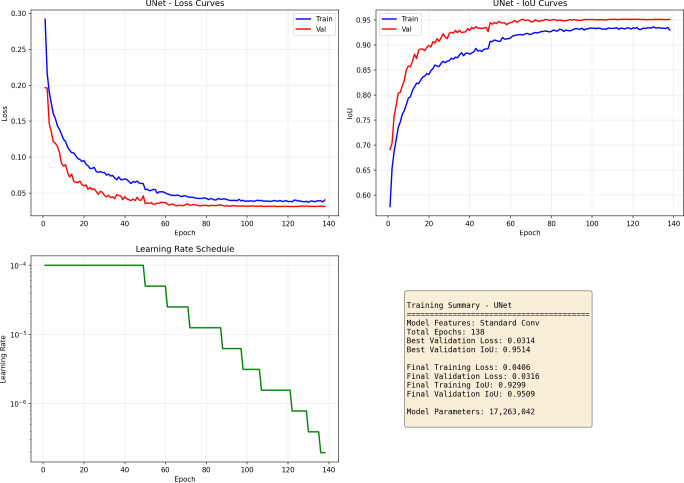
Training curves and learning rate schedule of UNet on SPVD-SEG.

**Figure 8:**
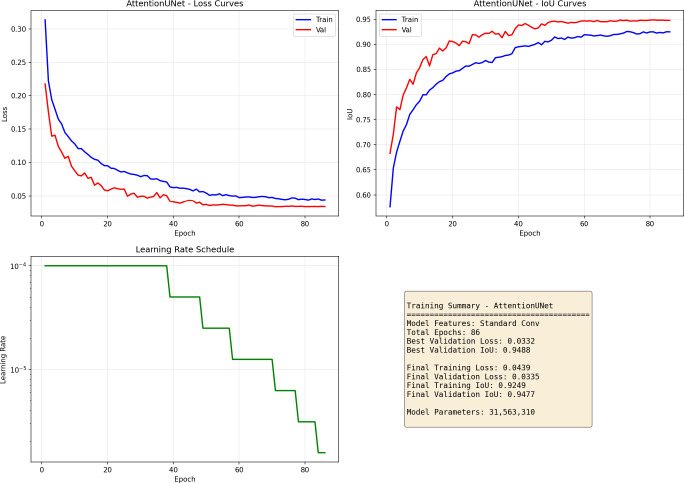
Training curves and learning rate schedule of AttentionUNet on SPVD-SEG.

**Figure 9:**
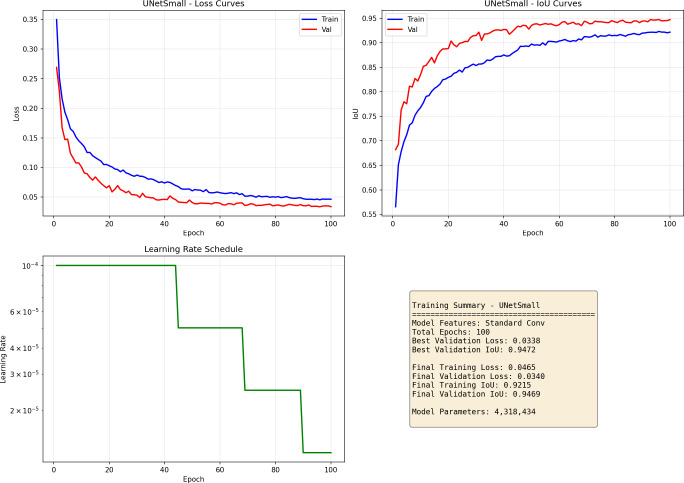
Training curves and learning rate schedule of UNetSmall on SPVD-SEG.

**Figure 10:**
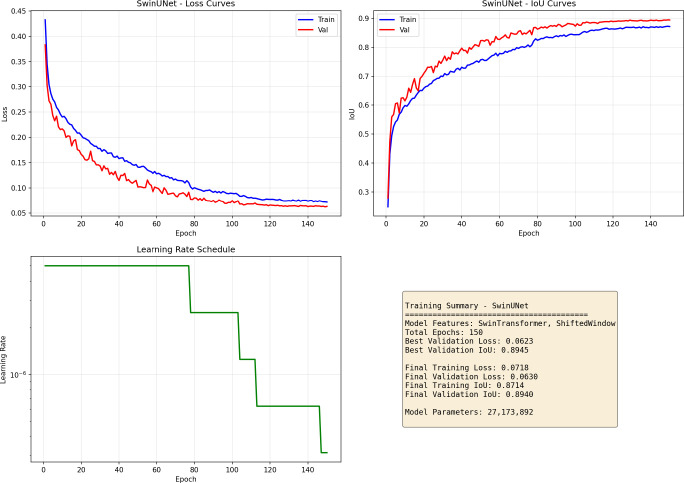
Training curves and learning rate schedule of SwinUNet on SPVD-SEG.

**Figure 11:**
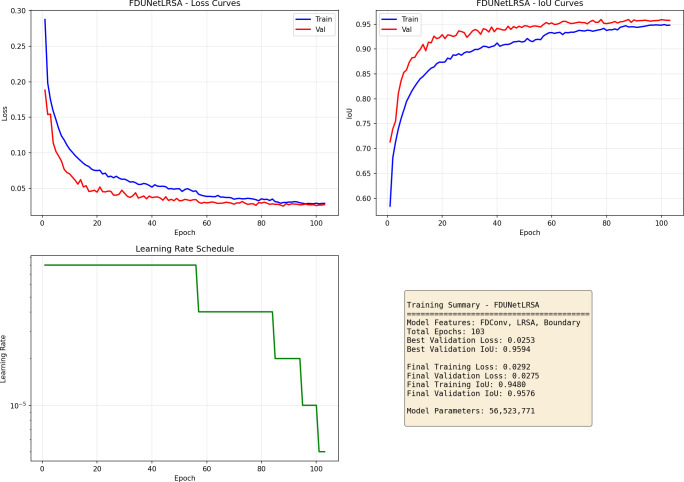
Training curves and learning rate schedule of FDUNetLRSA on SPVD-SEG.

The training curves of different segmentation models are presented in **Figures 7**–**11**.

- As shown in **Figure 7**, the classical UNet architecture performs reliably on the SPVD-SEG dataset, achieving a validation IoU of approximately 0.95. The smooth training curves demonstrate the dataset’s suitability for segmentation tasks.
- As shown in **Figure 8**, AttentionUNet demonstrates stable convergence during training. The validation IoU gradually increases and stabilizes around 0.95, while the training and validation loss decrease consistently. This indicates that the SPVD-SEG dataset provides sufficient supervision for learning accurate lesion boundaries.
- As shown in **Figure 9**, UNetSmall provides a lightweight alternative with significantly fewer parameters. Despite its reduced complexity, the model still achieves a validation IoU close to 0.95, indicating that the dataset supports efficient training of lightweight segmentation networks.
- As shown in **Figure 10**, The transformer-based SwinUNet achieves competitive segmentation performance, with validation IoU approaching 0.89. Although slightly lower than CNN-based models in this experiment, the model exhibits stable convergence and strong feature representation ability.
- As shown in **Figure 11**, Compared with AttentionUNet, this model achieves a slightly higher validation IoU of 0.959, demonstrating stronger capability in capturing fine-grained lesion structures. The learning rate scheduling also contributes to stable optimization during later training stages.

### 5.2 Visualization results analysis

**Figure 12:**
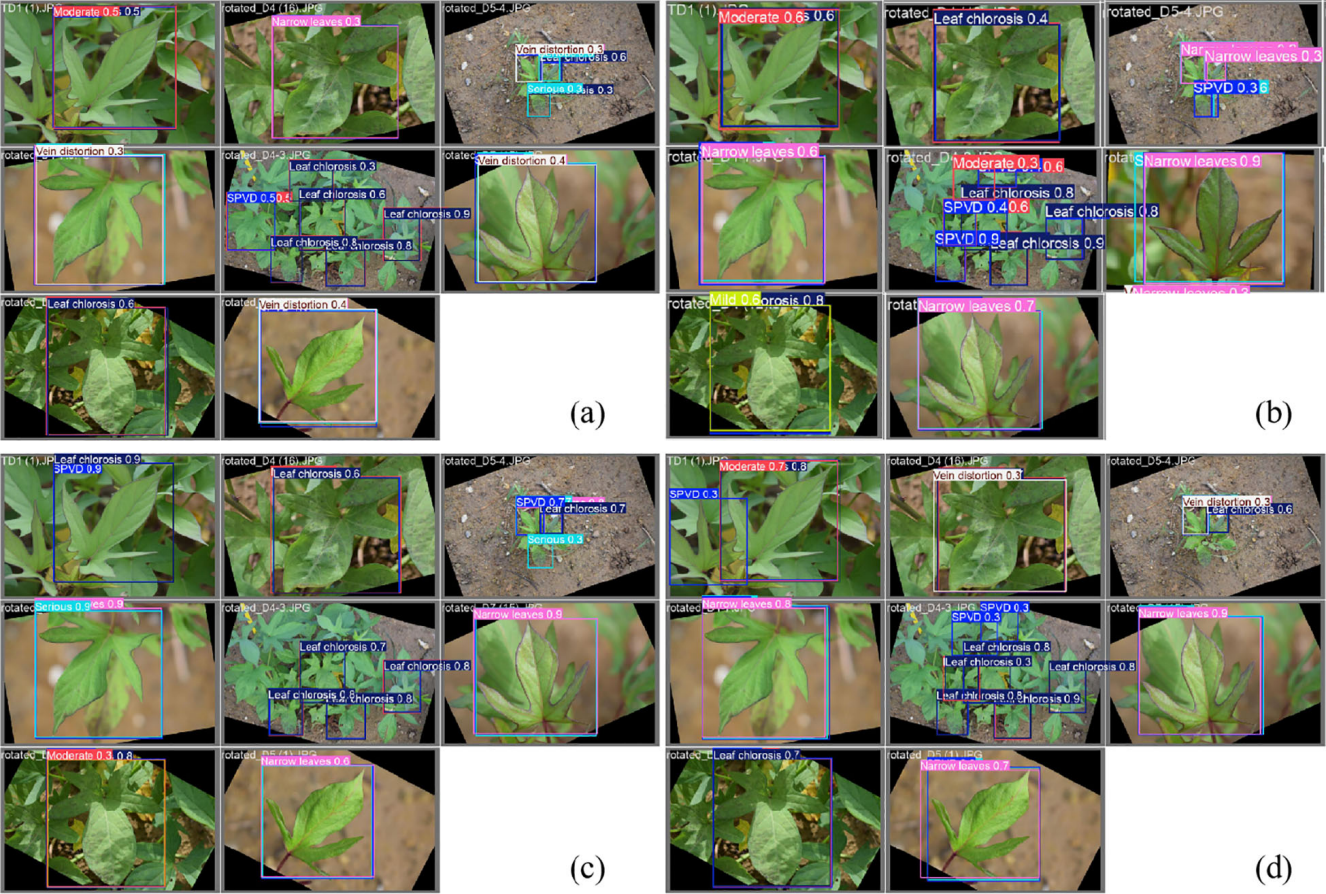
Detection results on real-field images.

As shown in **Figure 12**, the models are able to successfully identify multiple SPVD-related symptoms under complex field conditions. The detection results demonstrate that the dataset contains diverse symptom patterns, including vein distortion, leaf chlorosis, narrow leaves, and different disease severity levels, which can be effectively learned by modern object detection models.

Despite variations in scale, background clutter, and partial occlusion, most disease regions are correctly localized with reasonable confidence scores. The visualization results also show that multiple symptoms can coexist within a single plant, highlighting the importance of multi-class detection capability in practical SPVD diagnosis.

**Figure 13:**
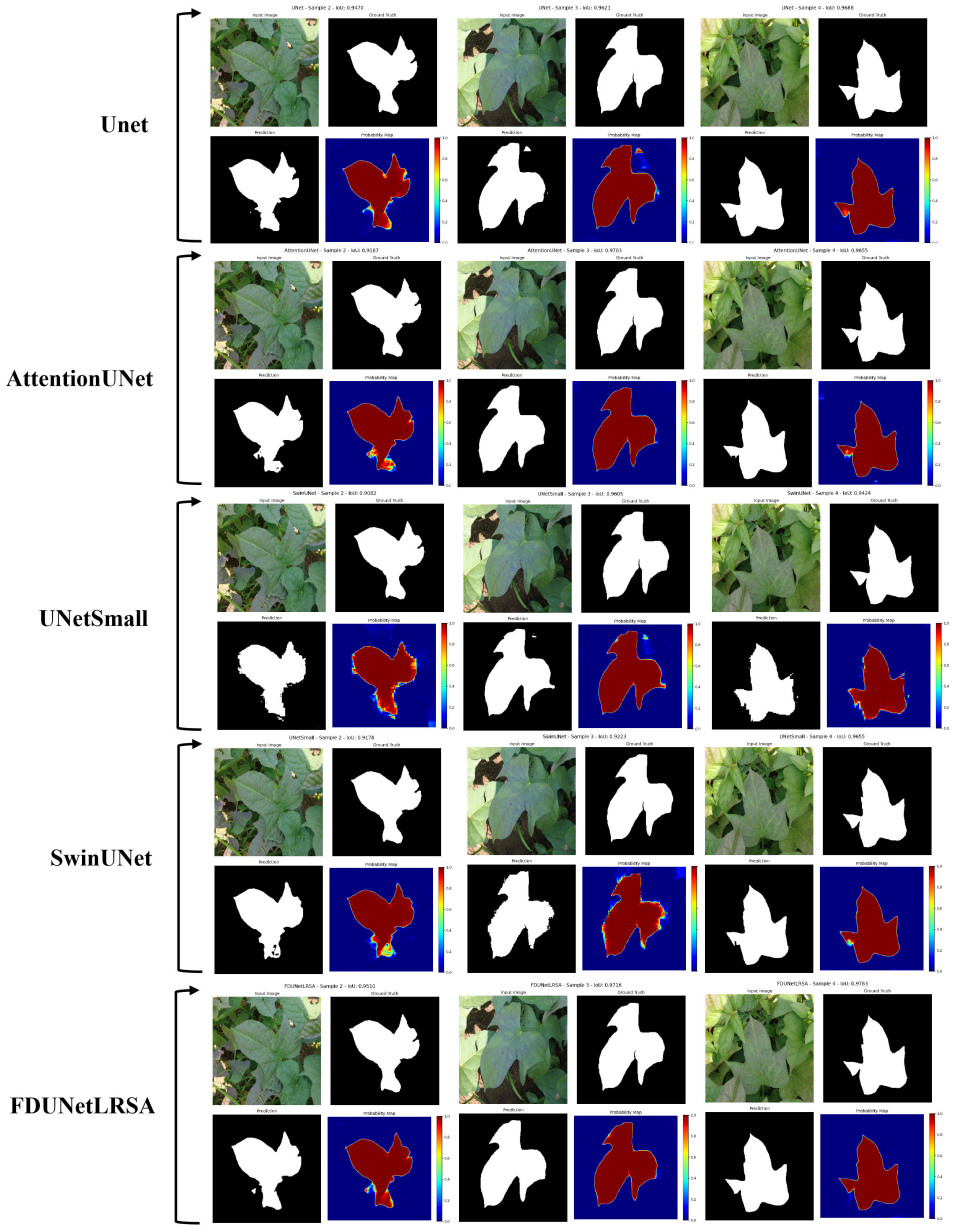
Qualitative comparison of segmentation results on the SPVD-SEG.

To further validate the effectiveness of our proposed framework, we provide a qualitative visualization of the segmentation results in **Figure 13**. As illustrated, the model effectively distinguishes the chlorotic spots and distorted leaf areas characteristic of SPVD from the complex soil and foliage backgrounds. Compared with the ground truth, the predicted masks exhibit high spatial consistency, particularly in capturing the fine-grained boundaries of early-stage lesions. These binary segmentation images supplement the quantitative metrics, providing a more intuitive demonstration of the dataset’s challenge and the model’s robust performance in real-field scenarios.

Overall, these qualitative results complement the quantitative evaluations presented earlier and further confirm that the proposed SPVD dataset provides reliable annotations, diverse symptom patterns, and realistic field conditions, making it suitable for benchmarking detection algorithms in agricultural disease diagnosis.

## 6 Discussion

This work makes several key contributions to SPVD-related agricultural vision research.

First, we introduce SPVD-Field, a task-oriented and multi-task dataset suite, where the detection and segmentation subsets were independently collected under different acquisition settings and annotation objectives. Unlike existing datasets that focus on a single task or controlled environments, SPVDField explicitly captures real-world variability, including scale, viewpoint, illumination, and background complexity.

Second, we propose a task-oriented dataset design paradigm by organizing the dataset into two complementary sub-datasets, SPVD-DET and SPVD-SEG. This design preserves task-specific data quality while maintaining semantic consistency, enabling flexible usage for detection, segmentation, and multi-task learning.

Third, we establish a unified evaluation framework with standardized data splits and baseline benchmarks, facilitating reproducible research and fair comparison across different methods.

Beyond its value for computer vision benchmarking, the SPVD-Field dataset also provides practical relevance for agricultural disease monitoring and crop management. SPVD is known for its complex symptom manifestation, which may vary depending on infection severity, plant developmental stage, environmental stress, and co-infection conditions. Early symptoms are often visually ambiguous and difficult to distinguish from nutrient deficiency or environmental stress, increasing the difficulty of reliable field diagnosis.

The dataset captures multiple physiological manifestations of SPVD under natural cultivation conditions, including chlorosis, vein distortion, leaf narrowing, and irregular lesion patterns. These symptom variations reflect realistic disease progression and provide valuable visual references for developing robust disease diagnosis systems. In practical agricultural scenarios, accurate early detection of SPVD can support timely intervention, reduce disease spread, and improve crop management efficiency. Therefore, the proposed dataset not only serves as a benchmark for detection and segmentation algorithms, but also contributes to future intelligent plant protection and precision agriculture applications.

These contributions collectively provide a practical and extensible foundation for future research on SPVD detection, segmentation, and related agricultural vision tasks.

### 6.1 Task-oriented dataset design

The SPVD-Field dataset was developed to address the scarcity of publicly available datasets for SPVD research under realistic agricultural conditions. Existing plant disease datasets are often limited to classification tasks or controlled imaging environments, which restrict their applicability to field deployment scenarios. In contrast, SPVD-Field provides a task-oriented dataset suite designed to support both symptom detection and lesion segmentation, enabling multi-level visual analysis of SPVD symptoms.

A notable characteristic of the dataset is its multi-task structure, consisting of SPVD-DET and SPVDSEG subsets. Detection annotations enable plant-level symptom localization suitable for large-scale field monitoring, while segmentation annotations provide pixel-level lesion information that supports finegrained analysis of disease patterns. This complementary design allows the dataset to serve as a unified benchmark for different computer vision tasks related to SPVD diagnosis.

### 6.2 Realistic field characteristics

Another key aspect of SPVD-Field is that the images were collected directly under real field conditions, introducing challenges such as background clutter, illumination variations, occlusion, and scale diversity. These characteristics better reflect practical agricultural environments compared with laboratory-style datasets. As a result, the dataset provides a more realistic benchmark for evaluating the robustness and generalization ability of vision-based disease detection models.

The SPVD-Field dataset provides a realistic benchmark for evaluating both accuracy and efficiency trade-offs. It can support future research on lightweight model design, real-time inference, and deployment on edge devices such as mobile platforms and agricultural robots.

The dataset also captures multiple SPVD symptom categories, including vein distortion, leaf chlorosis, narrow leaves, and different severity levels. The coexistence of multiple symptoms within individual plants increases the complexity of visual diagnosis and highlights the importance of multi-class detection and fine-grained symptom analysis. Such characteristics make the dataset suitable for evaluating advanced architectures, including hybrid CNN–Transformer models designed to handle small or subtle disease patterns.

### 6.3 Limitations and future directions

Despite these advantages, several limitations should be acknowledged. First, the current version of SPVD-Field is based on RGB imagery only. While RGB images are widely used and easily accessible in practical applications, they may not fully capture physiological or spectral characteristics required for precise disease severity estimation. Incorporating multi-modal data, such as multispectral or hyperspectral imagery, would further enhance the dataset’s capability for fine-grained disease analysis.

Second, although the dataset supports detection and segmentation tasks, explicit annotations for disease severity levels are limited. Future extensions of this work could include severity grading annotations or continuous severity estimation labels to support more advanced phenotyping studies. In addition, SPVD symptom expression may vary across cultivars, environmental conditions, and infection stages, which remains a challenge for robust cross-region disease generalization.

Overall, SPVD-Field provides a realistic and task-oriented benchmark for SPVD research. By supporting both detection and segmentation tasks under field conditions, the dataset facilitates the development and evaluation of robust computer vision models for intelligent plant disease monitoring.

## 7 Conclusions

The SPVD-Field dataset is a comprehensive multi-source benchmark designed to support SPVD detection and segmentation under real agricultural conditions. By providing standardized training, validation, and testing splits together with baseline benchmark results, the dataset enables reproducible, fair, and comparable evaluations of computer vision models. Its emphasis on complex field variability, including illumination changes, occlusion, scale differences, and background interference, makes it highly representative of practical agricultural scenarios. In addition, the inclusion of both detection and segmentation annotations supports multi-granularity analysis of SPVD symptoms, contributing to the advancement of agricultural vision research and facilitating the development of robust, field-deployable plant phenotyping systems.

Furthermore, SPVD-Field provides a valuable foundation for future studies on intelligent crop monitoring and precision agriculture. Future work will explore joint detection–segmentation frameworks, multi-task learning strategies, and lightweight deployment-oriented models for real-time field applications. The dataset may also be extended with disease severity annotations, temporal monitoring data, and multimodal information to support more comprehensive SPVD assessment and large-scale agricultural monitoring systems.

## Data Availability

The datasets presented in this study can be found in online repositories. The names of the repository/repositories and accession number(s) can be found below: https://dx.doi.org/10.21227/hq1q-jp43.
